# LncRNA HCP5 Induces Gastric Cancer Cell Proliferation, Invasion, and EMT Processes Through the miR-186-5p/WNT5A Axis Under Hypoxia

**DOI:** 10.3389/fcell.2021.663654

**Published:** 2021-06-11

**Authors:** Ming Gao, Liying Liu, Yudan Yang, Mengyi Li, Qingqing Ma, Zhiwei Chang

**Affiliations:** ^1^Department of Oncology, The First Affiliated Hospital of Zhengzhou University, Zhengzhou, China; ^2^Department of Medical Record, The First People’s Hospital of Zhengzhou, Zhengzhou, China

**Keywords:** hypoxia, gastric cancer, lncRNA HCP5, miR-186-5p, WNT5A

## Abstract

**Objective:**

To experimentally determine the involvement and mechanism of long non-coding RNA (lncRNA) HCP5 in the development of gastric cancer (GC).

**Methods:**

Detection of HCP5, miR-186-5p, and WNT5A expression in clinical GC tissues and adjacent healthy tissues was performed, followed by Pearson correlation analysis. BGC-823 and AGS cells, with interferences of HCP5, miR-186-5p, and WNT5A, were cultured under hypoxia. MTT, colony formation assay, Caspase-3 activity assay, and transwell assay were applied for the determination of cell proliferation, viability, apoptosis, and invasion, respectively. Expressions of WNT5A and protein markers of epithelial-mesenchymal transition (EMT) in cells were detected by western blotting. And the binding of HCP5 and WNT5A to miR-186-5p was validated using dual-luciferase reporter assay.

**Results:**

In GC tissues, an increase in HCP5 and WNT5A expressions and a reduction in miR-186-5p expression were found, and the negative correlation between miR-186-5p and HCP5/WNT5A was proven. Subsequently, under hypoxia, an increase in HCP5 and WNT5A expressions and a decrease in miR-186-5p expression in GC cells were confirmed. In addition, in GC cells under hypoxia, the inhibition of HCP5 suppressed cell biological activity and EMT, while the inhibition of miR-186-5p or the overexpression of WNT5A led to the opposite changes.

**Conclusion:**

An upregulation of WNT5A expression by HCP5 competitively binding to miR-186-5p promotes GC cell development.

## Introduction

Gastric cancer (GC), as a common malignancy of the digestive tract, has a high incidence, high mortality, and poor prognosis (Hashad et al., 2016). Globally, GC incidence ranks the fifth among tumors, and its mortality ranks the third (Bray et al., 2018). Smoking, alcohol consumption, and other unhealthy habits are risk factors for the development of GC (Guo et al., 2009). At present, the main treatments for GC are surgical resection and adjuvant therapy. In adjuvant therapy, the poor efficacy and side effects of conventional antitumor drugs, alongside the difficult development of targeted drugs, are the main problems (Choi et al., 2015). Since early symptoms of GC are atypical, being similar to those of benign peptic ulcer, GC patients are frequently assigned advanced or late tumor stage at the time of diagnosis (Guo et al., 2009). Patients with late GC often miss the chance of radical surgical treatment and are at risk of postoperative recurrence and metastasis, emphasizing the importance of early diagnosis of this disease (Necula et al., 2019). Currently, upper gastrointestinal endoscopy, imageological examination, and tumor markers are the main diagnostic methods of GC. Among them, the first two methods have limitations, including high pricing, invasiveness, and limited detection capacity (Akahoshi et al., 2018). On the other hand, tumor markers as a non-invasive examination have a good prospect in achieving early diagnosis (Duffy et al., 2014).

Long non-coding RNAs (lncRNAs), length >200 nt, often directly or indirectly involve in the development of various diseases by pre- and post-transcriptional, epigenetic, or other regulation (Zhao J. et al., 2018). Increasing studies have found that lncRNA can be used as markers for tumor development. For example, Zhang Y. X. et al. (2018) have shown that invasion of lung cancer can be promoted by lncRNA TUC338 via activating the MAPK pathway. García-Venzor et al. (2019) have revealed that breast cancer cell viability can be promoted by lncRNA HAL. In addition, lncRNAs can function as competing endogenous RNA (ceRNA) by competitively binding to microRNAs (miRNAs), thus regulating downstream signaling pathways. MiRNAs, as a class of non-coding RNAs (∼22 nt), can specifically target the 3′UTR of target mRNAs, thereby inhibiting mRNA translation (Zhang E. et al., 2018). Many studies have confirmed the involvement of lncRNA in tumor development via the regulation of miRNAs. For example, in breast cancer, lncRNA HOTAIR has been proved to affect cell growth, migration, invasion, and apoptosis through the miR-20a-5p/HMGA2 axis (Zhao W. et al., 2018). The regulation of miR-331-3p and miR-338-3p by lncRNA XLOC006390 can promote occurrence and metastasis of cervical cancer (Luan and Wang, 2018). There are also studies on the relationship between lncRNA and GC development. For example, lncRNA SNHG16 has been proved to downregulate DKK3 expression, encouraging epithelial-mesenchymal transition (EMT) in GC (Zhou et al., 2019); lncRNA SNHG17 has been proven to promote the progression of GC by epigenetic silencing of p15 and p57 (Zhang et al., 2019).

LncRNA HCP5 (HCP5) is a human-specific regulatory lncRNA, participating in adaptive and innate immune responses (Kulski, 2019). Recent studies have shown the association between regulation of miRNAs by HCP5 and progression of various cancers. For example, Yu et al. (2018) have found that HCP5 regulates MACC1 to promote cervical cancer development by inhibiting miR-15a. Liang et al. (2018) confirm that HCP5 promotes thyroid follicular carcinoma progression through miRNAs sponge. And HCP5 can also act as a sponge of miR-4656, performing regulation of CEMIP and promotion of proliferation of prostate cancer cells (Hu and Lu, 2020). However, the role and mechanism of HCP5 in GC under hypoxia have not been studied. Therefore, we investigated the function of HCP5 in GC based on the *in vitro* cell culture under hypoxia, with the goal to discover a novel diagnostic biomarker and a new treatment target.

## Materials and Methods

### Tissue Specimens

Sixty samples were collected from GC patients diagnosed and treated at the First Affiliated Hospital of Zhengzhou University between 2016 and 2019. The samples were separated into two groups: the GC group (tumor tissue) and the normal group (adjacent histologically normal tissue). Based on the tumor stage, GC tissues were further classified into I, II, III, and IV groups. Based on the degree of tumor differentiation, GC tissues were divided into the poorly differentiated group and the well-differentiated group. This study was approved by the ethics committee of the First Affiliated Hospital of Zhengzhou University and received informed consent from each patient, with strict adherence to the Declaration of Helsinki.

### Cell Culture and Cell Transfection

Human normal gastric epithelial cell line GES-1 and human GC cell lines (MKN45, HGC-27, BGC-823, MGC-803, and AGS) were purchased from American Type Culture Collection (ATCC). All cell lines were grown in a DMEM culture medium (Gibco, United States) containing 10% fetal bovine serum (FBS, Gibco, United States) in a 37°C incubator with 5% CO_2_.

BGC-823 and AGS cells were transfected with miR-186-5p mimics or mimics NC, and miR-186-5p inhibitor or inhibitor NC; samples were classified as the miR-186-5p group, the oe-NC group, the in-miR-186-5p group, and the in-NC group, respectively. These two cell lines with no transfection, or with transfection of negative siRNA, HCP5 siRNA, siRNA-HCP5 + in-miR-186-5p, and siRNA-HCP5 + pcDNA-WNT5A, were cultured under hypoxic conditions, 1% O_2_, 5% CO_2_, or 94% N_2_ for 48 h; hypoxic samples were classified as 1% O_2_, 1% O_2_ + si-NC, 1% O_2_ + si-HCP5, 1% O_2_ + si-HCP5 + in-miR-186-5p, and 1% O_2_ + si-HCP5 + WNT5A groups. BGC-823 and AGS cells cultured under normoxia condition were classified as the 20% O_2_ group. All cell transfection was performed using Lipo2000 (Invitrogen, United States) according to the manufacturer’s instruction. MiR-186-5p mimics and inhibitor were obtained from the GenePharm (Shanghai, China).

### qRT-PCR

Following total RNA extraction from the tissues with TRIzol (Sigma-Aldrich, Germany), reverse transcription was performed using a random primer reverse transcription kit (Thermo, United States). The SYBRGREEN kit (TaKaRa, Japan) was applied for detecting respective mRNA expression, with GAPDH or U6 as an internal reference. A total of six replicates were set up for qRT-PCR assay. Quantitative expressions of target genes were calculated using the 2^–△^^△^^Ct^ method. Table 1 presents the primer sequences used.

**TABLE 1:** Primer sequences.

RNA	Sequences (5′ to 3′)
lncRNA HCP5	F: 5′-CCGCTGGTCTCTGGACACATACT-3′
	R: 5′-CTCACCTGTCGTGGGATTTTGC-3′
miR-186-5p	F: 5′-AAGAATTCTCCTTTTGGGCT-3′
	R: 5′-GTGCGTGTCGTGGAGTCG-3′
WNT5A	F: 5′-ATTCTTGGTGGTCGCTAGGTA-3′
	R: 5′-CGCCTTCTCCGATGTACTGC-3′
GAPDH	F: 5′-CATCACTGCCACCCAGAAGACTG-3′
	R: 5′-ATGCCAGTGAGCTTCCCGTTCAG-3′
U6	F: 5′-TCTGCTCCTATCCCAATTACCTG-3′
	R: 5′-ACTCCCGGATCTCTTCTAAGTTG-3′

### MTT Assay

Treated cells in the logarithmic growth phase were inoculated into 96-well plates (2,000 cells/well) and cultured for 24, 48, and 72 h. After that, the cells were cultured for another 4 h with 20 μL of 5 mg/mL MTT solution (Sigma-Aldrich, Germany). After the solution aspirated, 150 μL of DMSO (Sigma-Aldrich, Germany) was added, and the solution was shaken for 15 min. A microplate reader was employed to measure the absorbance at 570 nm of each well.

### Colony Formation Assay

After digestion with 0.25% trypsin, the cells were inoculated into 6-well plates (1 × 10^3^ cells/well) and fully dispersed, followed by incubation at 37°C with 5% CO_2_. When colonies were formed in culture dishes, cells were rinsed with PBS twice, followed by 15-min methanol fixation. After Giemsa stain, photography was performed under an inverted microscope for counting the number of colonies.

### Caspase-3 Activity Assay

The supernatant of the treated BGC-823 and AGS cells was removed. Precooled PBS buffer was used to rinse the cells three times and removed. Next, after cell lysis was performed, the lysis buffer was collected for centrifugation (4°C, 8,000 r/min for 10 min), and the supernatant was extracted to a new tube. Caspase-3 assay kit (ab270770, Abcam, United States) was used to assess Caspase-3 activity in each group of cells and to detect cell apoptosis.

### Transwell Invasion Assay

The Matrigel (BD Biosciences, United States)-coated transwell upper chamber contained 100 μL of cell suspension, while the lower chamber with 700 μL of the medium contained 20% fetal bovine serum. After 12–24 h of culture at 37°C with 5% CO_2_, transwell inserts were removed. PBS was employed to rinse the inserts three times, and then fixation with 1% glutaraldehyde was performed. Following rinsing and drying, samples were stained using 0.1% crystal violet for 12 h. After rising and drying again, 6–10 fields were randomly observed under an upright microscope. Positive cells in each field were counted, and three randomly selected fields were photographed for later statistical analysis.

### Dual-Luciferase Reporter Assay

Using a pmirGLO dual-luciferase miRNA expression vector (Promega, United States), HCP5 wild-type (HCP5-WT), mutant (HCP5-MUT), WNT5A wild-type (WNT5A-WT), and mutant (WNT5A-MUT) plasmids were constructed. MiR-186-5p mimics and HCP5-WT/MUT plasmids or WNT5A-WT/MUT plasmids were cotransfected into 293T cells by Lipo2000 (Invitrogen). Finally, after 48-h transfection, a dual-luciferase reporter assay kit was utilized to measure luciferase activity.

### Western Blotting

Extraction of proteins using a cell lysis buffer was followed by the determination of protein concentration with a BCA kit (ab102536, Abcam, United States). After the proteins were separated with SDS-PAGE electrophoresis, they were blotted onto PVDF membranes, which were blocked with 5% non-fat dry milk for 1 h. Membranes were incubated overnight with primary antibodies WNT5A (ab179824, Abcam, United States), E-cadherin (ab40772, Abcam, United States), vimentin (ab92547, Abcam, United States), N-cadherin (ab98952, Abcam, United States), Snail1 (ab216347, Abcam, United States), ZEB (ab203829, Abcam, United States), and β-actin (ab8226, Abcam, United States) at 4°C and were rinsed three times with PBS afterward. Membranes were incubated for 1 h with secondary antibodies at room temperature and were rinsed for another three times with PBS. Proteins were developed by a chemiluminescence reagent and then photographed with a gel imaging system. ImageJ software was used for the analysis of gray values of protein bands. Relative quantification of protein was carried out with β-actin as an internal reference.

### Statistics Analysis

One-way analysis of variance and independent samples *T*-test were carried out using SPSS 26.0. The data were presented as mean ± standard deviation (SD). Pearson correlation analysis was also performed. A *p* < 0.05 indicated a statistically significant difference.

## Results

### Upregulation of lncRNA HCP5 Expression in GC and Hypoxia-Induced Promotion of HCP5 Expression

**FIGURE 1:**
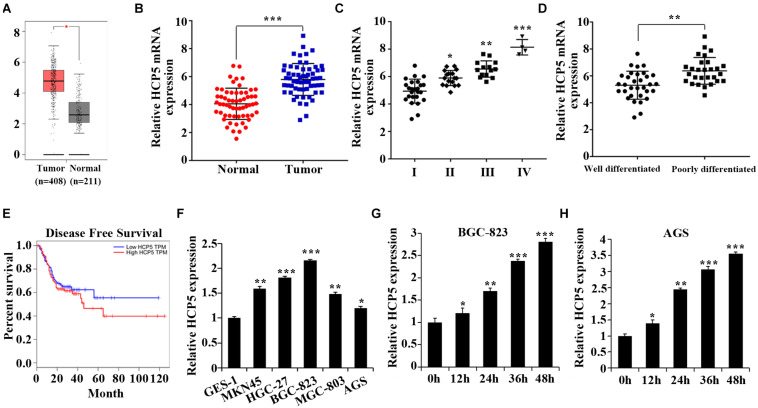
Upregulation of HCP5 expression in gastric cancer and hypoxia-induced promotion of HCP5 expression. **(A)** TCGA database results of HCP5 expression in gastric cancer (GC) tissues and adjacent tissues. **(B)** qRT-PCR-based detection of HCP5 expression in GC tissues and adjacent tissues; ***p < 0.001 vs. normal group. **(C)** qRT-PCR-based detection of HCP5 expression in GC tissues in different stages; *p < 0.05, **p < 0.01, ***p < 0.001 vs. Group I. **(D)** qRT-PCR-based detection of HCP5 expression in GC tissues with different degree of differentiation; **p < 0.01 vs. well-differentiated group. **(E)** TCGA database results of disease-free survival of GC patients with high or low expression of HCP5. **(F)** qRT-PCR analysis of HCP5 expression in normal gastric epithelial cells (GES-1) and GC cells (MKN45, HGC-27, BGC-823, MGC-803, and AGS; *p < 0.05, **p < 0.01, ***p < 0.001 vs. GES-1 cells. **(G,H)** qRT-PCR analysis of HCP5 expression in BGC-823 and AGS cells with induction of 1% O_2_ for 0–48 h; *p < 0.05, **p < 0.01, ***p < 0.001 vs. 0 h group.

We first found that HCP5 was highly expressed in GC tissues from The Cancer Genome Atlas (TCGA) database^1^ (Figure 1A). This result was further verified by the qRT-PCR results of GC tissues (Figure 1B). And the expression of HCP5 increased with the progression of GC stage; an obvious increase in HCP5 expression was also revealed in the poorly differentiated group (Figures 1C,D). The TCGA database also indicated a lower disease-free survival in GC patients with highly expressed HCP5 (Figure 1E). In addition, the expression of HCP5 was noticeably higher in 5 GC cell lines compared to GES-1 cells (Figure 1F). Among all GC cell lines, BGC-823 cells had the relatively highest HCP5 expression while AGS the lowest. Compared with that under normoxia, HCP5 expression was significantly elevated under hypoxia, with the relatively highest expression in the BGC-823 and AGS cells after 48 h of hypoxia exposure (Figures 1G,H).

### Inhibition of Hypoxia-Induced Proliferation, Invasion, and EMT Process by Downregulation of lncRNA HCP5

**FIGURE 2:**
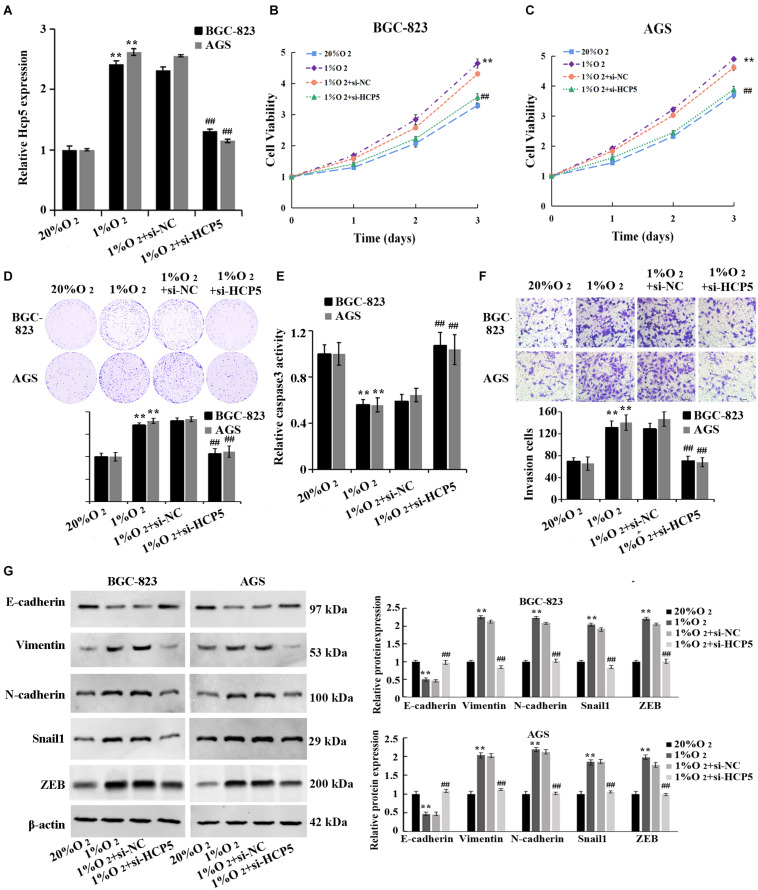
Inhibition of hypoxia-induced proliferation, invasion, and EMT process in gastric cancer cells by downregulation of HCP5. **(A)** qRT-PCR-based detection of transfection efficiency of HCP5 siRNA; **(B,C)** MTT-based detection of cell proliferation. **(D)** Colony formation assay-based detection of cell viability. **(E)** Caspase-3 activity in each group. **(F)** Transwell assay-based detection of cell invasion. **(G)** Western blotting-based detection of expression of E-cadherin, vimentin, N-cadherin, Snail1, and ZEB. **p < 0.01 vs. 20% O_2_ group; ^##^*p* < 0.01 vs. 1% O_2_ + si-NC group.

For the purpose of verifying the HCP5 function in hypoxia-induced GC, HCP5 siRNA was transfected into BGC-823 and AGS cells followed by qRT-PCR to determine transfection efficiency (Figure 2A). Hypoxia induced significant increases of GC cell proliferation, viability and invasion, and a decrease in apoptosis; however, these hypoxia-induced effects could be interfered HCP5 expression (Figures 2B–F). In addition, western blotting results (Figure 2G) showed that in BGC-823 and AGS cells, hypoxia induced a significant decrease in E-cadherin protein expression and an increase in vimentin, N-cadherin, Snail1, and ZEB. These hypoxia-induced effects could also be inhibited by HCP5. Collectively, these results proved that hypoxia could induce GC cell growth, while HCP5 could inhibit GC cell proliferation and metastasis.

### HCP5 Regulated miR-186-5p Expression That Targeted WNT5A Expression

**FIGURE 3:**
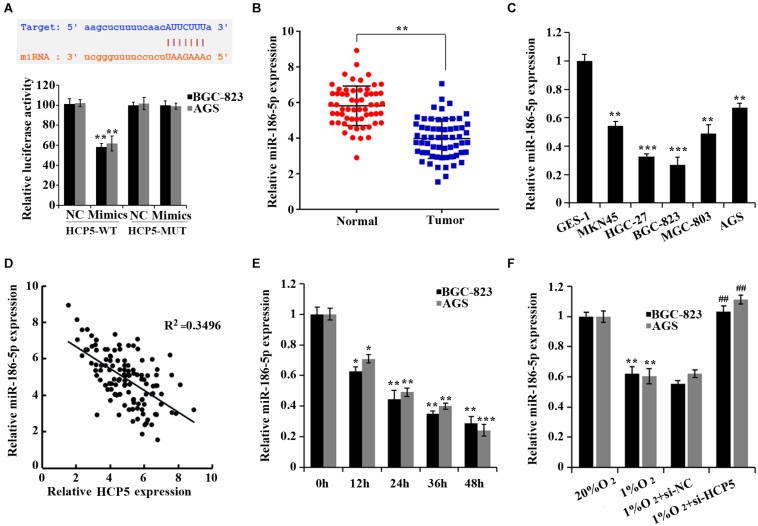
HCP5 acts as a miR-186-5p sponge. **(A)** Dual-luciferase assay-based validation of the targeting relationship between HCP5 and miR-186-5p; **p < 0.05 vs. NC group. **(B)** qRT-PCR-based detection of miR-186-5p expression in gastric cancer tissues and matched normal counterparts; **p < 0.01. **(C)** qRT-PCR-based detection of HCP5 expression in cells; *p < 0.05, **p < 0.01, ***p < 0.001 vs. GES-1 cells. **(D)** Pearson correlation analysis of HCP5 and miR-186-5p in gastric cancer tissues. **(E)** qRT-PCR analysis of HCP5 expression in BGC-823 and AGS cells at different time points of hypoxia; *p < 0.05, **p < 0.01, ***p < 0.001 vs. 0 h group. **(F)** qRT-PCR analysis of miR-186-5p expression in BGC-823 and AGS cells, **p < 0.01 vs. 20% O_2_ group; ^##^*p* < 0.01 vs. 1% O_2_ + si-NCgroup.

To determine the mechanism of HCP5 in regulating GC, a potential binding site of HCP5 to miR-186-5p was found in the starbase database^2^ (Figure 3A). And cotransfection of miR-186-5p mimics and HCP5-WT led to an apparent reduction in luciferase activity, suggesting an interaction between HCP5 and miR-186-5p (Figure 3A). Moreover, the expressions of miR-186-5p in the GC tissues and GC cell lines were significantly reduced (Figures 3B–C). Pearson correlation analysis further showed that HCP5 was negatively correlated to the expression of miR-186-5p in GC tissues (Figures 3D, *R*^2^ = 0.3496). In addition, a significant decrease in miR-186-5p in the hypoxia-treated GC cells was detected (Figure 3E), while an increase in cells was observed with the interference of HCP5 expression (Figure 3F).

**FIGURE 4:**
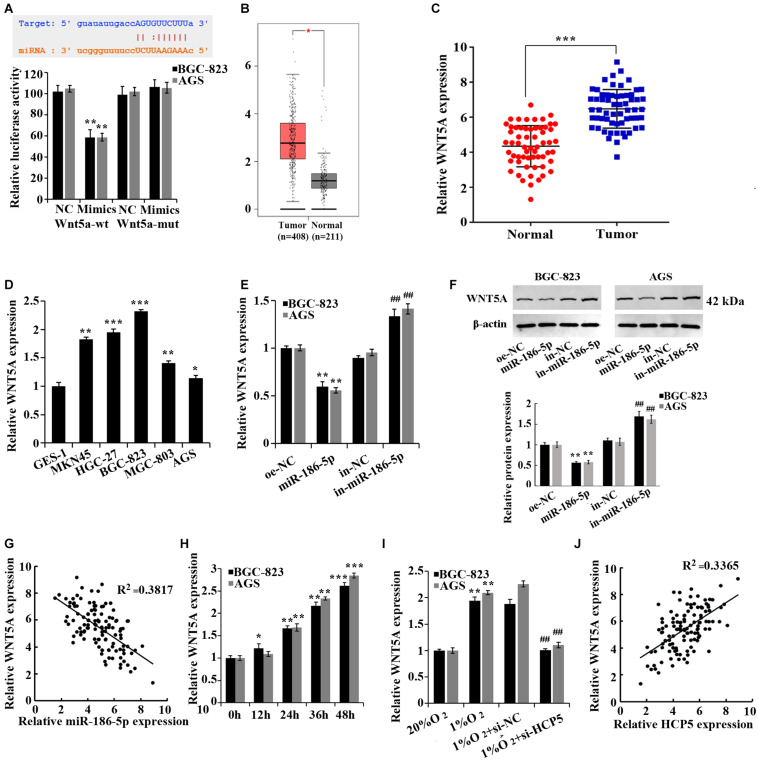
miR-186-5p targets and regulates WNT5A. **(A)** Dual-luciferase assay for validation of the target relationship between miR-186-5p and WNT5A; **p < 0.01. **(B)** WNT5A expression in gastric cancer (GC) tissues and matched normal counterparts shown in the TCGA database. **(C)** qRT-PCR-based detection of WNT5A expression in GC tissues and matched normal counterparts; ***p < 0.001. **(D)** qRT-PCR-based detection of HCP5 expression in cells; *p < 0.05, **p < 0.01, ***p < 0.001 vs. GES-1 cells. **(E)** qRT-PCR-based detection of WNT5A expression in cells; **p < 0.01 vs. oe-NC group; ^##^*p* < 0.01 vs. in-NC group. **(F)** Western blotting-based detection of WNT5A protein expression in cells. **p < 0.01 vs. oe-NC group; ^##^*p* < 0.01 vs. in-NC group. **(G)** Pearson correlation analysis of miR-186-5p and WNT5A expression in GC tissues (*n* = 60, *R*^2^ = 0.3817, *p* < 0.0001). **(H)** qRT-PCR analysis of WNT5A expression in BGC-823 and AGS cells at different time points of hypoxia; *p < 0.05, **p < 0.01, ***p < 0.001 vs. 0 h group. **(I)** qRT-PCR-based detection of WNT5A expression in BGC-823 and AGS cells; **p < 0.01 vs. 20% O_2_ group; ^##^*p* < 0.01 vs.1% O_2_ + si-NC group. **(J)** Pearson correlation analysis of HCP5 and WNT5A expression in GC tissues (*n* = 60, *R*^2^ = 0.3365, *P* < 0.0001).

Moreover, according to the prediction by the TargetScan database^3^, WNT5A was a target gene of miR-186-5p, and the binding of the two was confirmed by dual-luciferase assay (Figure 4A). The TCGA database (Figure 4B) and qRT-PCR (Figure 4C) confirmed that WNT5A was upregulated in GC tissues. We further confirmed that the mRNA level of WNT5A was increased in the GC cell lines (Figure 4D). However, a noticeable decrease in WNT5A expression in GC cells was observed after overexpressing miR-186-5p, while an increase in expression was observed after the interference of miR-186-5p (Figures 4E,F). Pearson correlation analysis showed that in GC tissues, WNT5A was negatively correlated to miR-186-5p (Figure 4G, *R*^2^ = 0.3817). Overall, miR-186-5p could target and regulate WNT5A expression in GC cells.

In addition, an elevation of WNT5A expression was observed in hypoxia-treated GC cells (Figure 4H). The interference of HCP5 expression induced a reduction of WNT5A expression in GC cells under hypoxia, while the overexpression of HCP5 expression had the opposite effect (Figure 4I). Pearson correlation analysis revealed that in GC tissues, HCP5 was positively correlated with WNT5A (Figure 4J, *R*^2^ = 0.3365). Collectively, HCP5 could competitively bind with miR-186-5p to upregulate WNT5A expression in GC cells.

### Regulation of Proliferation, Invasion, and EMT Process of Hypoxia-Treated Gastric Cancer Cells by HCP5 Through the miR-186-5p/WNT5A Axis

**FIGURE 5:**
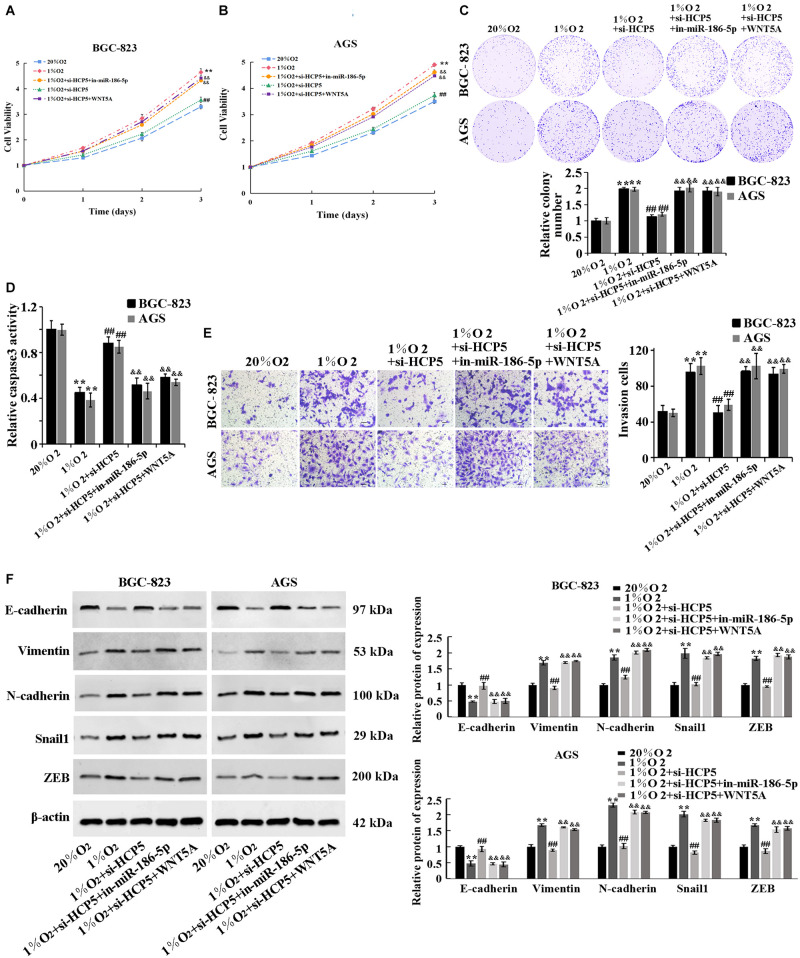
Regulation of proliferation, invasion, and EMT process of hypoxia-treated gastric cancer cells by HCP5 through the miR-186-5p/WNT5A axis. **(A,B)** MTT-based detection of cell proliferation. **(C)** Colony formation assay-based detection of cell viability. **(D)** Caspase-3 activity in each group. **(E)** Transwell-based detection of cell invasion. **(F)** Western blot-based detection of protein expression of E-cadherin, Vimentin, N-cadherin, Snail1, and ZEB. **p < 0.01 vs. 20% O_2_ group; ^##^*p* < 0.01 vs. 1% O_2_ group; ^&&^*p* < 0.01 vs. 1% O_2_ + si-HCP5 group.

To determine the involvement of miR-186-5p/WNT5A in the regulation of GC by HCP5, here we performed the cell rescue experiments. The results showed that there was a significant promotion of proliferation (Figures 5A,B), viability (Figure 5C), invasion (Figure 5E), and EMT process (Figure 5F) of BGC-823 and AGS cells, and a significant reduction in apoptosis (Figure 5D) in the 1%O_2_ + si-HCP5 + in-miR-186-5p group, compared with the 1% O_2_ + si-HCP5 group. The collective evidence supported that the promotion of GC cell proliferation and metastasis by low expression of HCP5 could be counteracted by transfection of the miR-186-5p inhibitor; transfection of WNT5A overexpression plasmids had similar effects.

## Discussion

Hypoxia is one of the important elements of the tumor microenvironment. Hypoxia can induce the proliferation, migration, infiltration, and drug resistance of tumor cells. In GC, the gastric mucosa is often under hypoxia (Payne and Bowen, 1981), resulting in the rapid growth of tumor tissues, disorder of neovascularization, and distant tumor metastasis, thus finally leading to the chemoresistance of GC tissue and cancerization. According to the results of our experiments, both in regular GC tissues and tissues under hypoxia, HCP5 was highly expressed. As the GC stage became higher, HCP5 expression in tissue rose. These results suggest the involvement of HCP5 and its role as a potential biomarker in GC development. This conclusion is similar to that proposed by Chen et al. (2020). Furthermore, inhibition of proliferation, invasion, and EMT and promotion of apoptosis in hypoxia-treated GC cells as a result of downregulating HCP5 were confirmed in our study. The inhibition of GC cell activity could also be achieved by downregulating other lnRNAs. For example, both lncRNA SNHG7 (Pei et al., 2021) and lncRNA HOXC-AS3 (Zhang E. et al., 2018), which were highly expressed in GC, can inhibit GC progression. Therefore, we speculate that by downregulating HCP5 in GC tissues, the progression of GC can be attenuated.

LncRNA usually functions as a molecular sponge for miRNAs. MiR-186-5p being a target of HCP5 was confirmed by dual luciferase reporter assay and qRT-PCR. We also found a downregulation of miR-186-5p in GC and its negative correlation with HCP5. Under hypoxia, GC cell proliferation, invasion, and EMT by a low expression of HCP5 could be counteracted after the interference of miR-186-5p expression. Liu et al. (2020) similarly found that miR-186-5p expression was downregulated in GC tissues and that miR-186-5p knockdown reversed the biofunctional effects and glycolytic activation following circ-NRIP1 silencing in GC cells.

In addition, we further studied the downstream target of miR-186-5p, and bioinformatics analysis predicted that WNT5A was one target gene of miR-186-5p. WNT5A is an important signaling molecule in various developmental processes, and its abnormal expression during development can lead to diseases, including tumors and bone degeneration (Pongracz and Stockley, 2006). The association between elevated expressed WNT5A and progression of melanoma (Weeraratna et al., 2002) and lung cancer (Wang et al., 2017), breast cancer (Prasad et al., 2018), and prostate cancer (Lee et al., 2018) was reported. And the reduction in WNT5A expression can significantly reduce the proliferation and EMT of GC cells (Hu et al., 2018). Here, prediction of WNT5A as a target of miR-186-5p was confirmed by dual-luciferase assay. We found the markedly increased WNT5A in both clinical GC tissues and hypoxia-treated GC cells, and WNT5A was positively correlated with HCP5. Subsequently, under the hypoxia condition, the upregulation of WNT5A expression could significantly counteract the inhibition of the biological functions and EMT of GC cells by a low expression of HCP5. These results suggested that HCP5 could upregulate WNT5A by binding to miR-186-5p, thereby leading to the promotion of proliferation, viability, and EMT of GC cells.

In existing studies, lncRNA H19 has been reported to upregulate WNT signaling activity by regulating miR-29b, thus promoting the EMT of colorectal cancer cells (Ding et al., 2018). He et al. (2017) also found that in non-small cell lung cancer, lncRNA FEZF1-AS1 can inhibit E-cadherin and regulate the WNT pathway to enhance the EMT. It can be concluded that WNT5A signaling is the key to cell development, and target genes such as WNT5A promote the function of lncRNAs in oncogenesis. Therefore, highly expressed lncRNAs, their targeted miRNAs, and the downstream target genes are potential target sites for cancer therapy.

## Conclusion

In summary, GC and hypoxia can induce high expression of HCP5, which can downregulate miR-186-5p and upregulate WNT5A, promoting proliferation, viability, invasion, and EMT and inhibiting the apoptosis of GC cells. Therefore, HCP5 can be a promising biomarker for GC. However, to fully elucidate the function of lncRNA HCP5, further research including animal model trials is needed prior to the clinical application of HCP5.

## Data Availability Statement

The original contributions presented in the study are included in the article/supplementary material, further inquiries can be directed to the corresponding author/s.

## Ethics Statement

The studies involving human participants were reviewed and approved by The First Affiliated Hospital of Zhengzhou University. Written informed consent for participation was not required for this study in accordance with the national legislation and the institutional requirements. This study was approved by the Medical Ethics Committee of The First Affiliated Hospital of Zhengzhou University.

## Author Contributions

MG and LL contributed to conception and design of the study, wrote the first draft of the manuscript, and wrote sections of the manuscript. YY and QM organized the database. ML and ZC performed the statistical analysis. All authors contributed to manuscript revision, read, and approved the submitted version.

## Conflict of Interest

The authors declare that the research was conducted in the absence of any commercial or financial relationships that could be construed as a potential conflict of interest.

## Footnotes

1 http://gepia2.cancer-pku.cn/#analysis

2 http://starbase.sysu.edu.cn/

3 http://www.targetscan.org
